# Magnetic Particle Imaging: An Emerging Modality with Prospects in Diagnosis, Targeting and Therapy of Cancer

**DOI:** 10.3390/cancers13215285

**Published:** 2021-10-21

**Authors:** Zhi Wei Tay, Prashant Chandrasekharan, Benjamin D. Fellows, Irati Rodrigo Arrizabalaga, Elaine Yu, Malini Olivo, Steven M. Conolly

**Affiliations:** 1 Institute of Bioengineering and Bioimaging, Agency for Science, Technology and Research (A*STAR), 11 Biopolis Way, #02-02 Helios Building, Singapore 138667, Singapore; malini_olivo@ibb.a-star.edu.sg; 2 Department of Bioengineering, 340 Hearst Memorial Mining Building, University of California Berkeley, Berkeley, CA 94720-1762, USA; prashantc@berkeley.edu (P.C.); bdfello@berkeley.edu (B.D.F.); irati.rodrigo@berkeley.edu (I.R.A.); elaineyu@berkeley.edu (E.Y.); sconolly@berkeley.edu (S.M.C.)

**Keywords:** magnetic particle imaging, magnetic nanoparticles, magnetic hyperthermia, magnetic drug delivery

## Abstract

**Simple Summary:**

Magnetic Particle Imaging (MPI) is an emerging imaging technique that provides quantitative direct imaging of superparamagnetic iron oxide nanoparticles. In the last decade, MPI has shown great prospects as one of the magnetic methods other than Magnetic Resonance Imaging with applications covering cancer diagnosis, targeting enhancement, actuating cancer therapy, and post-therapy monitoring. Working on different physical principles from Magnetic Resonance Imaging, MPI benefits from ideal image contrast with zero background tissue signal, enabling hotspot-type images similar to Nuclear Medicine scans but using magnetic agents rather than radiotracers. In this review, we discussed the relevance of MPI to cancer diagnostics and image-guided therapy as well as recent progress to clinical translation.

**Abstract:**

Background: Magnetic Particle Imaging (MPI) is an emerging imaging modality for quantitative direct imaging of superparamagnetic iron oxide nanoparticles (SPION or SPIO). With different physics from MRI, MPI benefits from ideal image contrast with zero background tissue signal. This enables clear visualization of cancer with image characteristics similar to PET or SPECT, but using radiation-free magnetic nanoparticles instead, with infinite-duration reporter persistence in vivo. MPI for cancer imaging: demonstrated months of quantitative imaging of the cancer-related immune response with in situ SPION-labelling of immune cells (e.g., neutrophils, CAR T-cells). Because MPI suffers absolutely no susceptibility artifacts in the lung, immuno-MPI could soon provide completely noninvasive early-stage diagnosis and treatment monitoring of lung cancers. MPI for magnetic steering: MPI gradients are ~150 × stronger than MRI, enabling remote magnetic steering of magneto-aerosol, nanoparticles, and catheter tips, enhancing therapeutic delivery by magnetic means. MPI for precision therapy: gradients enable focusing of magnetic hyperthermia and magnetic-actuated drug release with up to 2 mm precision. The extent of drug release from the magnetic nanocarrier can be quantitatively monitored by MPI of SPION’s MPS spectral changes within the nanocarrier. Conclusion: MPI is a promising new magnetic modality spanning cancer imaging to guided-therapy.

## 1. Introduction

**Figure 1:**
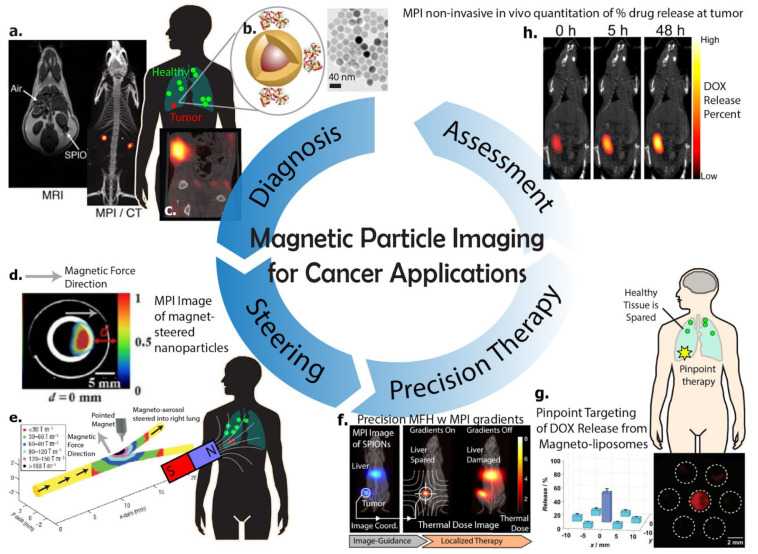
Overview figure for Magnetic Particle Imaging’s prospects in diagnosis, targeting, and therapy of cancer. Magnetic Particle Imaging (MPI) is an emerging magnetic imaging technology that works completely differently from MRI, providing radiation-free tracer-like contrast and linear quantitation with nanogram sensitivity to superparamagnetic iron-oxide nanoparticles (SPIONs). While most MPI research is still preclinical, MPI hardware has recently reached clinical scale scanners and is en route to clinical translation. (**a**) Zheng et al. 2016 [8]—Comparison of MRI SPION contrast to MPI contrast of the same SPIONs. (**b**) Arami et al. 2017 [11] — schematic of cancer-targeted magnetic nanoparticles. (**c**) Yu et al. 2017 [12]—MPI image of SPIONs accumulated within a tumor. (**d**) Banura et al. 2017 [13]—magnetic steering of magneto-aerosol with MPI as post-event verification. (**e**) Dames et al. 2007 [14]—magnetic tip for targeted delivery of magnetic aerosol to lung. (**f**) Tay et al. 2018 [15]—MPI scanner’s gradients enable pinpoint heating at user-selected locations, heating SPIONs at the tumor without heating off-target SPIONs. (**g**) Liu et al. 2018 [16] — magnetic gradients enable pinpoint drug release at target location without triggering release from adjacent nanocarriers 2 mm away. (**h**) Zhu et al. 2019 [17] — MPI in vivo non-invasive quantification of the percentage of release of drug from nanocarriers in mice tumors, enabling real-time assessment of the success of drug delivery for cancer. Figures within insets reproduced with permission from respective authors cited in reference numbers and publishers.

Magnetic Particle Imaging (MPI) is an emerging magnetics-based imaging technique first introduced by Philips, Hamburg in 2005 [1]. While the name is very similar to Magnetic Resonance Imaging (MRI), it operates on very different physical principles. Unlike MRI, where the signal comes from the precession of nuclear spin magnetic moments of the target nuclei (e.g., 1H, 2H, 13C, 17O, 19F, 23Na, 31P), the MPI signal is obtained from the ensemble magnetization of superparamagnetic iron oxide nanoparticles (SPION) as described by the Langevin model [2]. Because there are no SPIONs found in native biological tissue unlike the ^1^H in water and biological tissue sensed by MRI, MPI benefits from zero tissue background signal and achieves excellent image contrast comparable to tracer images typical of nuclear medicine scans such as positron emission tomography (PET) or single-photon emission computerized tomography (SPECT), which are the gold standard for diagnostic cancer imaging [3,4,5]. Since only SPIONs produce signal in an MPI scan, the MPI images obtained are fully quantitative in a linear fashion and are robust to minute changes in susceptibility. In comparison, the same SPIONs in an MRI scan are typically semi-quantitative as they produce contrast changes via susceptibility differences (Figure 1a), yielding a non-linear indirect effect on the ^1^H signal [2]. MPI operates in the kilohertz frequency range where magnetic fields fully penetrate tissue, bone, and air with negligible attenuation and reflection differences. Thus, MPI does not have any view limitations and works robustly even in lungs [6,7,8,9] and bones, which are challenging for MRI [2,10] and ultrasound.

Besides the excellent image contrast, one of the other key benefits of Magnetic Particle Imaging for cancer imaging is the relatively high sensitivity of the modality. The electronic magnetization of SPIONs sensed by MPI is 22 million times stronger than that of the nuclear magnetization of water (^1^H) at 7 Tesla [2]. Furthermore, the dose limit of iron oxide is 510 mg according to Lu et al. 2010, which is 25 million times higher than the 2 ng dose limit of PET [18,19]. The 2 ng value was calculated from the 370 MBq FDA-mandated dose limit for 18-FDG divided by the specific (radio)activity of 1ng of 18-fluorine (in MBq, averaged specific activity value). This implies that MPI can increase the systemic administered dosage to compensate and ensure tumor detection at 260 nM Fe sensitivity, even though this is poorer sensitivity than the 2 pM of PET [20]. Therefore, if SPIONs can achieve similar targeting efficiencies to tumors as 18-FDG, MPI can be expected to be competitive with PET on a dose-limited comparison, and thus help avoid radiation dose (especially important for pediatrics). Other advantages include the near-infinite signal half-life of SPIONs enabling longer time for circulation and binding to tumors while the 110 min half-life of 18-FDG necessitates a PET scan merely 40 min after injection [19]. Convenience is also improved as SPIONs can be used off-the-shelf, thus avoiding cyclotron facility overheads and the radiation safety measures for hot chemistry preparations. 

Regarding the imaging agent, the SPION magnetic “tracers” used in MPI will differ depending on the application. For stem cell or immune cell labeling, carboxydextran-based SPIONs have shown good labeling efficiency, likely due to the affinity of the dextran coating to cell uptake and internalization. For vascular imaging or tumor targeting, long-circulating stealth SPIONs with PEG-based coatings are ideal due to the enhancement of blood circulation half-life, allowing more time for the tracer to remain in circulation for vascular imaging or for the tracer to aggregate into the tumors. For magnetic hyperthermia applications where the magnetic nanoparticles are heated via external alternating magnetic fields (AMF), the magnetic core of the SPIONs must demonstrate high heating performance, i.e., good specific absorption rate (SAR) values at typical hyperthermia frequencies (100–1000 kHz) [21]. It is important to note that these qualities are not mutually exclusive and an SPION can be designed with multiple of these qualities such as a high heating performance magnetic core with stealth PEG-coating. 

## 2. Physical Mechanisms Underlying Magnetic Particle Imaging

In brief, MPI performs spatial encoding, signal detection, and image reconstruction based on very different magnetic principles from MRI. From the high-contrast and spatial resolution characteristics, MPI is more similar to PET and SPECT, although it uses non-radioactive SPIONs at 20–100 nm sizes rather than small-molecule radiotracers. This section will explain the mechanism of MPI and its spatial and temporal resolution. 

### 2.1. Localization and Collection of Signal from a Specific Slice or Volume

**Figure 2:**
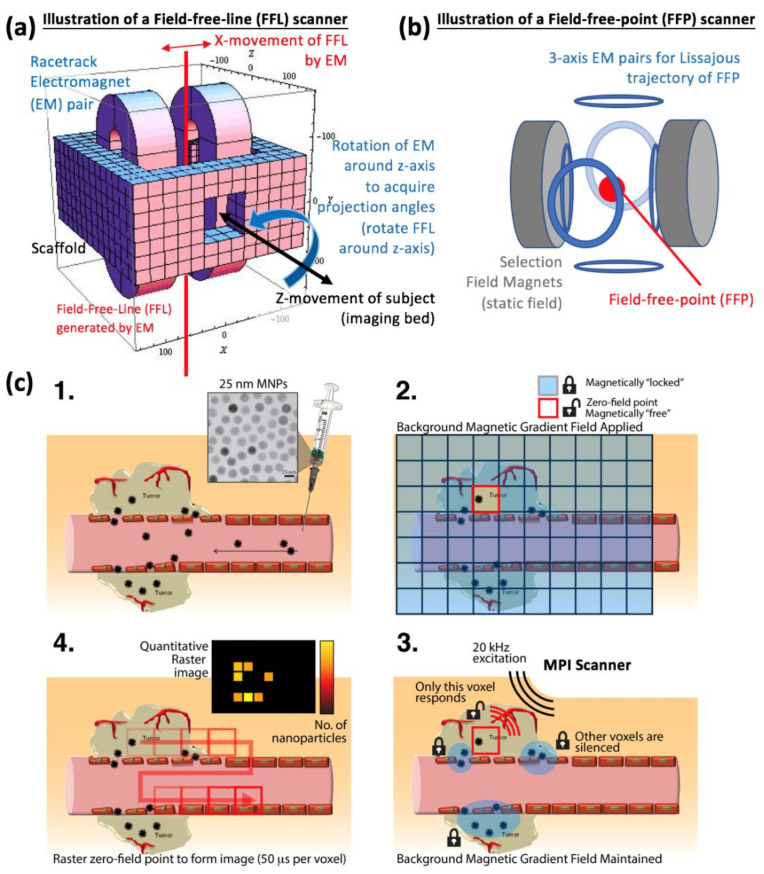
Physical mechanisms underlying how Magnetic Particle Imaging (MPI) scans and produces an image. (**a**) Scanner configuration for a field-free-line (FFL) MPI scanner. (**b**) Scanner configuration for a field-free-point (FFP) scanner. (**c**) Illustrative workflow diagram on how the previously defined MPI-related magnetic fields can be applied to SPIONs that have accumulated within a tumor in terms of how to spatially encode, signal detect, and image reconstruct the MPI image. The background tumor image is adapted from Jhaveri AM, Torchilin VP. Multifunctional polymeric micelles for delivery of drugs and siRNA. Front Pharmacol 2014; 5:77 under a CC by 3.0 creative commons license [22].

Magnetic Particle Imaging has two methods of localization of signal and thereby achieves spatial encoding in order to reconstruct an image. For the system matrix method, a point source of SPIONs is physically moved to every voxel in the field-of-view (FOV) and the MPI harmonic signal recorded as a calibration to determine the system matrix transfer function. In order to encode a different MPI harmonic signal at every voxel, a static background selection field is applied. The selection field is defined as a gradient field (magnetic field strength varies spatially) with a zero-field region at a central point (defined as field-free-point system—FFP) or a zero-field in a line geometry (defined as field-free-line system—FFL) as shown in Figure 2a,b. The differing background field strength as a function of position changes the SPION magnetization and results in a different MPI harmonic signal depending on position in the FOV. The static selection field alone cannot excite an MPI signal, and thus a time-varying drive field of 25 kHz and 16 mT is applied. The definition of a drive field is a monotonal excitation magnetic field operating in the kilohertz frequency range that aims to generate rapid magnetization changes in SPIONs as the MPI signal. When superimposed on top of the selection field, the result is the motion of the FFP in a Lissajous trajectory so as to pass near every voxel in the FOV at least once during the scan. In conclusion, spatial encoding for the system matrix meth-od uses the combination of the selection field gradient and a Lissajous (rather than raster) trajectory to determine a unique MPI harmonic signature for each and every voxel in the FOV [5]. 

For the x-space method, there is no pre-calibration step. Spatial encoding relies on the fact that SPIONs physically at the location of the FFP or FFL give the largest amount of signal and SPIONs away from the FFP give less and less signal in accordance to the point-spread-function (PSF) of that specific MPI scanner and SPION combination. This is basically predicated on the PSF as imaging spot size, with a smaller spot size enabling greater precision in discerning at-FFP signal and suppressing off-FFP signal. To acquire the entire FOV, the FFP or FFL is rastered across the FOV, usually in a cartesian trajectory, although the Lissajous trajectory used in the system matrix method could work too. The x-space drive field is usually in the same-axis as the detector coil (single-axis) and uses typical values of 20 kHz and 20 mT [3].

### 2.2. Signal Detection and Image Reconstruction Approach for MPI

MPI uses receive coils, defined as inductive solenoid or saddle-shaped wire coil sensors, which detect the SPION signal based off the time-varying magnetization changes of the SPION in response to the drive field. As a result, the signal strength is proportional to dM/dt and frequencies around 20 kHz are preferred as a trade-off between high signal strength from dM/dt and minimizing relaxation-induced blurring when the SPION cannot keep up with the drive field switching [3]. Unlike MRI where the readout timing is usually delayed after the excitation, the MPI signal is read out at the same time as the drive field (excitation) application. There is thus a large amount of direct feedthrough of the drive field into the inductive received signal. This is mitigated by high-pass or band-pass filters as well as gradiometric sensing coil design.

Image reconstruction for the system matrix method solves an inverse problem using the calibrated system function (3D matrix) achieved by recording the MPI harmonics at each and every voxel in the FOV. Image reconstruction for the x-space method relies on knowledge of the instantaneous position of the FFP and FFL in 3D space. The current instantaneous MPI signal is directly gridded to the known FFP/FFL location [3].

### 2.3. Spatial Resolution and Time Requirements for MPI

The current spatial resolution for MPI is around 0.5–2 mm depending on the magnetization characteristics of the SPION used as well as the gradient strength of the scanner [2]. Figure 2 compares the resolution and sensitivity of MPI to other imaging modalities. The temporal resolution of MPI can be relatively good at 45 frames per second as achieved by system matrix MPI due to the speed of the Lissajous trajectory [5]. 

## 3. Imaging Cancer Using Magnetic Particle Imaging

In brief, MPI is similar in image-quality to PET because of its zero-background, high-contrast, and ~1 mm spatial resolution. However, MPI’s imaging agent of SPIONs cannot utilize the Warburg effect to target tumors and must rely on other mechanisms such as targeting of cancer cell receptors or cancer-specific proteases. This section discusses the various imaging studies on cancer that have been performed with MPI. 

**Figure 3:**
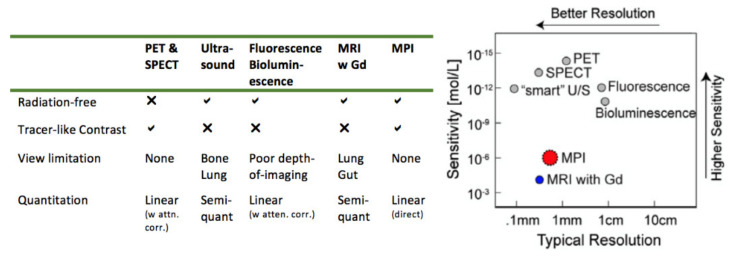
Comparison of MPI to other molecular imaging modalities. Figure on right panel adapted with permission from Saritas et al. J. Magn. Reson. 229 [2]. Copyright 2013 Elsevier.

The gold standard for clinical cancer imaging is Positron Emission Tomography (PET). The ability of 18-FDG to selectively accumulate in even small metastatic tumors due to the Warburg effect and the tracer-nature and positive contrast of PET scans allow for unambiguous diagnosis of tumor presence and location [20]. Coverage of the whole-body is possible except for the brain or bladder due to the low contrast caused by high background 18-FDG uptake by healthy tissue. However, PET scans still have a non-negligible radiation dose and are not recommended for pediatric imaging. MRI and CT are also widely used for cancer imaging but often require tumors to be relatively large for reliable detection on scans. In this context, Magnetic Particle Imaging is promising as it provides tracer-like contrast (see Figure 1a–c) without any radiation dose due to the use of magnetic “tracers” as opposed to radiotracers. Figure 3 summarizes the differences of MPI from other imaging modalities for cancer imaging. In practice however, the SPIONs used in MPI still need to make progress towards matching 18-FDG’s high affinity to cancerous tissue in order to be competitive with PET.

The earliest demonstrated application of Magnetic Particle Imaging towards cancer was a 2014 in vitro study by the University Hospital of Schleswig Holstein and Institute of Medical Engineering at the University of Luebeck, Germany [23]. Custom dextran-coated SPIONs (UL-D) were synthesized and demonstrated good internalization by head and neck squamous cancer cells as well as significant MPI signal via in vitro measurements using a Magnetic Particle Spectrometer. Although in vivo images were not demonstrated, the authors comprehensively characterized the labeled cells showing that their MPI-suitable SPIONs did not impact cell mitochondrial activity (MTT assay), cell viability (annexin V-APC-Propidium Iodide flow cytometry), cell proliferation (xCELLigence DP), cytokine secretion (Bead-based immunoassays for IL-6, IL-8, IL-1β and TNF-α), and reactive oxygen species generation (ROS assay by Dichlorofluorescein diacetate). These assays suggest that labeling of the cancer cells should not negatively impact tumor behavior such as increased tumor invasion or metastases.

In 2016, another in vitro study demonstrated the detection of cancer-specific proteases using changes in Magnetic Particle Spectrum (MPS) of MPI-compatible monodisperse iron oxide nanoparticles [24]. The linker-peptide-aggregated nanoparticles demonstrated a significant change in their spectrum when exposed to cancer-specific proteases. Although this assay was not verified for in vivo MPI, since MPI can be calibrated to tune specifically to a designated MPS (color MPI), this strategy could be promising to increase MPI image-specificity to cancer cells. Sensitivity can be improved by optimization of the magnetic core size [25] as well as designing contrast-enhancing MPI pulsed excitation rather than continuous-wave excitation [26].

**Figure 4:**
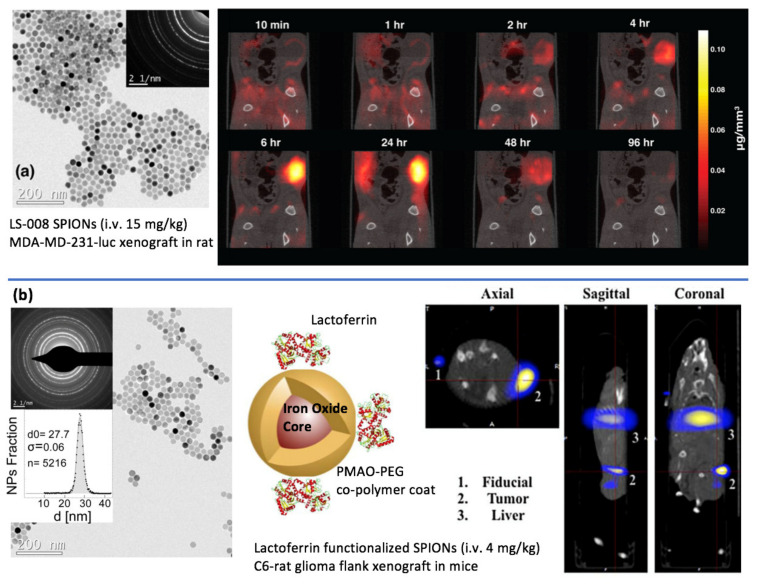
Imaging Cancer using Magnetic Particle Imaging. Figures adapted with permission from Yu et al. Nano Lett. 17. Copyright 2017 American Chemical Society. (**a**) Yu et al. 2017 [12] used LS-008 SPIONs injected i.v. at 15 mg/kg into an MDA-MD-231-luc flank xenograft in rat. The long-circulating SPIONs were non-targeted and after a few hours of systemic circulation, accumulated by the enhanced permeability and retention effect in the tumor. The image time course showcases the benefits of MPI with tracer-like contrast and direct linear quantitation, enabling clear visualization of the particle EPR dynamics with initial rim enhancement, accumulation, and then wash-out. (**b**) Arami et al. 2017 [11] used Lactoferrin functionalized SPIONs injected i.v. at 4 mg/kg for targeting of a C6-rat glioma flank xenograft in mice. After 2 h post-injection, the MPI image showed accumulation in the tumor together with substantial clearance to the liver. Figures reproduced from Arami et al. Nanoscale. 9 with permission from the Royal Society of Chemistry.

The earliest full study of in vivo Magnetic Particle Imaging of cancer (Figure 4a) was demonstrated in 2017 by the University of California Berkeley on their academic MPI scanner using long-circulating SPIONs (LS-008) from Lodespin Laboratories [12]. 

The cancer model used seven athymic nude rats bearing flank xenografts of MDA-MB-231-luc breast tumor cells. This work emphasized some of the inherent advantages of MPI for cancer imaging such as excellent image contrast and full quantitation of the tracer dynamics from administration to initial rim enhancement of the tumor, accumulation within the tumor between 1–24 h (peaking at 6 h), and then slow clearance to the liver over a period of 96 h. The signal-to-background ratio of the tumor was very high (>100) as there was no background uptake of SPIONs by biological tissue unlike 18-FDG. Because attenuation correction and signal half-life compensation is not required in MPI, the image quantification was demonstrated to be facile and straightforward. The tracer accumulation in the tumor occurred via enhanced permeability and retention effect (EPR) as this was an untargeted SPION study without cancer-targeting functionalization.

Later in 2017, the first in vivo MPI image of cancer using targeted SPIONs (Figure 4b) was demonstrated by the Stanford School of Medicine Department of Radiology in mice [11]. This study improved targeting to the flank xenograft of C6 brain cancer cells via surface functionalization with lactoferrin and also by placing a permanent magnet on the rodent flank. The SPIONs were multi-modal with Cy5.5 NIRF and 67-Ga radiolabel for near-infrared and SPECT imaging respectively. This study further demonstrated the excellent image contrast of MPI as compared with near-infrared imaging and showed that it approaches the image contrast achievable by 67-Ga SPECT images.

### Imaging Cell Therapy for Cancer Immunotherapy Using Magnetic Particle Imaging

In brief, MPI has many advantages for monitoring of labeled adoptive cell transfer immune cells such as long-lasting magnetic label that does not lose signal over time by radioactive decay and MPI’s high-contrast yet quantitative nature. This section introduces cancer immunotherapy and recent efforts to image immunotherapy with MPI. 

In the last twenty years, immunotherapy for cancer has steadily gained traction in clinical practice. There are five major types of cancer immunotherapy: (1) cancer vaccines, (2) cytokine therapies, (3) adoptive cell transfer (ACT), (4) immune checkpoint inhibitors, and (5) oncolytic virus therapies [27,28,29,30,31]. Of all these categories, MPI is well-poised to contribute in the adoptive cell transfer category and oncolytic virus category. This is because the magnetic nanoparticles used in MPI have optimal core sizes of 20–30 nm and can thus label immune cells (micron-sized) or oncolytic viruses (150–240 nm). For the adoptive cell transfer category, there is a need to verify that the transferred cells have arrived at and remain at the target cancer site throughout the course of therapy. Furthermore, it is essential to monitor the viability and functionality of the cells to ensure the success of the therapy [32]. These requirements are similar to the imaging requirements for stem cell therapy. Since MPI has been validated in many stem cell therapy studies [33,34,35,36,37], we anticipate MPI’s advantages to be applicable to the adoptive cell therapy application as well. The main benefits of MPI in stem cells are innately transferable to the adoptive cell therapy application, such as (1) no loss in signal over time from magnetic cell labels enabling >90% of signal left over 89 days in vivo [33], (2) no radiation dose that will limit the length of a longitudinal study, (3) direct and quantitative measurement of magnetic label that is unaffected by changes in subject anatomy background over time [34], and (4) potential for assessment of viability of labeled cells via color MPI spectroscopic techniques demonstrated in various MPI studies that leverages microenvironment sensitivity for color/contrast change or for multi-contrast multiplexing [38,39,40,41]. These initial stem cell studies have demonstrated that the magnetic label remains internalized within the cell population of interest, and that any released label is rapidly cleared to the liver and does not confound the quantitation [33].

ACT has shown the greatest success in “liquid” malignancies such as B lymphocyte leukemia and lymphoma. However, ACT as an immunotherapy for solid tumors has been hampered by an inability to adequately manipulate infused T cells to efficiently traffic into and specifically target deep-seated tumors for destruction, while minimizing immune-related adverse events (irAEs) caused by low-level recognition of antigen on surrounding healthy tissues [42]. Clinicians thus require real-time information on the biodistribution of ACT products in patients for accurate prognosis and treatment success [43]. MPI of SPION-labeled ACT immune cells can provide high-contrast, sensitive visualization of biodistribution and are thus ideal for this unmet need. The same SPIONs also appear on MRI scans (albeit lower contrast), thus allowing MPI’s quantitative nature to complement the high-resolution anatomic MRI scans [44,45]. Rivera-Rodriguez et al. recently demonstrated MPI of ACT immune cells in a mouse model and showed that labeled immune cells showed up in the brain of C57BL/6 mice bearing intracranial KLuc-gp100 tumors 24 h after ACT infusion [46]. 

Furthermore, ideally immune cells should demonstrate native magnetic signal in order to prevent under-counting that occurs when in vitro magnetic labels are diluted by cell division. Recent efforts tried to overcome this limitation by genetically modifying cells with genes from magnetotactic bacteria [47,48,49], in order to produce magnetic crystals to enable label-free native magnetic contrast, but this has not been widely implemented on different mammalian cell types yet.

**Figure 5:**
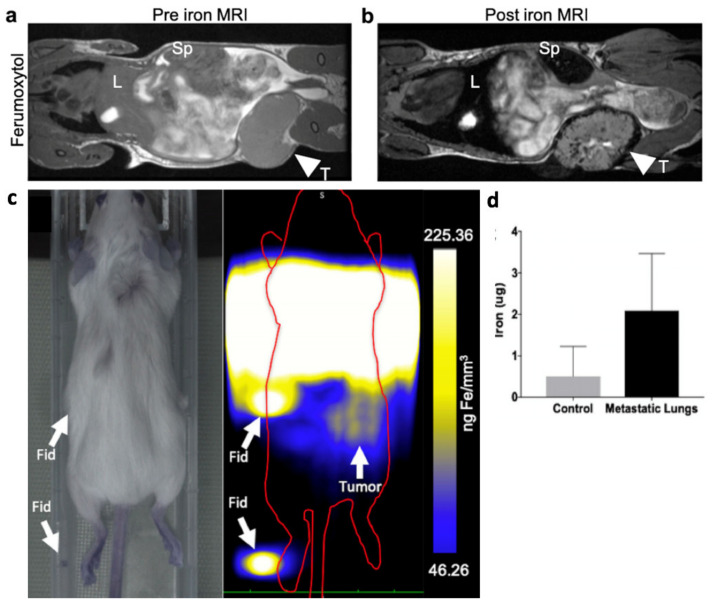
Imaging of Tumor-Associated Macrophages (TAMs) using MRI and MPI. Adapted with permission from Makela et al. Mol Imaging Biol 22. Copyright 2020 Springer Nature. [51]. (**a**) MRI image before non-targeted systemic i.v. injection of 0.5 mmol/kg Ferumoxytol (*n* = 8). L denotes liver, Sp denotes spleen, and T denotes tumor. (**b**) MRI image at 24 h post-injection of Ferumoxytol, where TAMs were seen to have iron uptake due to signal voids visible within the tumor. (**c**) MPI image at 24 h post-injection of Ferumoxytol. The image resolution was lower because Ferumoxytol is not optimal for MPI due to 7-fold worse spatial resolution of the nanoparticle than MPI standard Ferucarbotran. The MPI signal, while visible in the tumor, was not visible in the lung, as the lungs could not be spatially resolved from the liver due to the poor Ferumoxytol spatial resolution. (**d**) Ex vivo MPI of the lungs showed a significantly higher amount of iron in lung metastases compared with healthy control, indicating the presence of iron-labeled TAMs. This study demonstrated that not all MRI iron contrast work well for MPI due to differences in the physical principles of the imaging signal between MRI and MPI.

Other than ACT, Magnetic Particle Imaging has also been demonstrated to be helpful in other immunology studies that help advance the field of cancer immunotherapy. For example, the tumor microenvironment is known to greatly impact the success rate and thus a better understanding will help decipher the mechanisms of immunotherapies, define predictive biomarkers, and identify novel therapeutic targets. Figure 5 showcases recent work on MPI to track tumor-associated macrophages (TAMs). [50,51] Aptly named “Magnetic Particle Imaging of Macrophages Associated with Cancer: Filling the Voids Left by Iron-Based Magnetic Resonance Imaging.”, the study showcased how MPI’s positive contrast and quantitative nature complements the traditional MRI images of TAMs. In addition to this, MPI can also image inflammation by in situ labeling of inflammatory immune cells [52]. Although this study did not target cancer cells per se, the same in situ labeling concept could be used to image the inflammatory tumor microenvironment. 

## 4. Magnetic-Based Steering and Targeting Strategies Using MPI Hardware

In brief, MPI use of the strongest magnetic gradients in the imaging field (up to 7 Tesla per meter) equips the MPI scanner to perform magnetic steering of magnetic agents to target tumor sites. This section elaborates on recent efforts to demonstrate this. 

**Figure 6:**
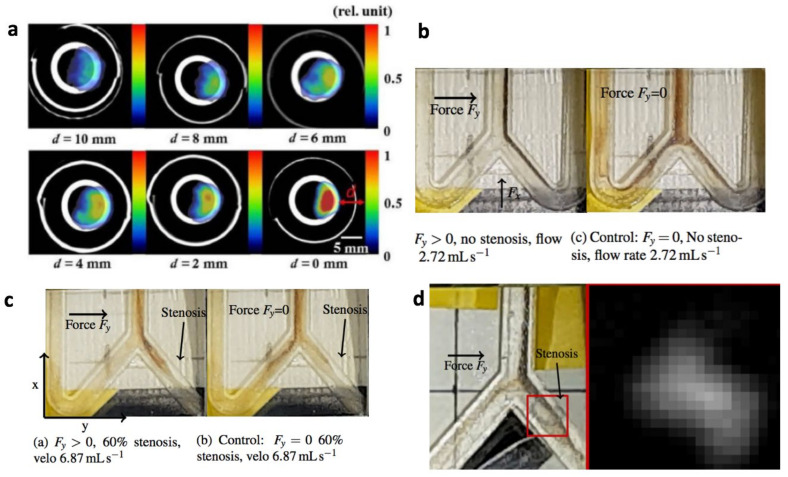
MPI image-guided magnetic steering of magnetic nanoparticles. Figures adapted with permission from Banura et al. Jpn. J. Appl. Phys 2017 [13] under Creative Commons 4.0. (**a**) Magnetic steering of aerosolized magnetic nanoparticles to deposit in a user-selected side of a lung imaging phantom. With closer distance d of the magnetic steering point to the lung, the stronger the accumulation of the magnetic nanoparticles. (**b**) Griese et al. 2020 [65] demonstrated magnetic steering of magnetic nanoparticles in a bifurcation flow phantom. Figures (**b**)–(**d**) adapted with permission Griese et al. J. Magn. Magn. Mater. 498. Copyright 2020 Elsevier. Without the magnetic steering in the control experiment, the dark brown nanoparticle stream bifurcated evenly. Once magnetic steering was turned on, 100% of the particles flowed into the selected right stream. (**c**) One important proof this study demonstrated was that magnetic steering can be performed against a strong flow rate of 6.87 mL/s, as shown by steering of particles into the right arm with a 60% stenosis, although the control clearly shows the flow rate favoring the left arm by order-of-magnitude. (**d**) Seamless switching between steer and image mode was shown in steering particles into an 100% stenosis arm, demonstrating that it is possible to maintain sufficient magnetic force to steer while performing a quick MPI image scan with a time ratio of 20:1 for steer:image.

To introduce this topic, we must first note that one of the key benefits of using a magnetic imaging agent is the fact that magnetism remains the strongest force-from-a-distance method for remote steering or targeting [53,54,55]. There have been many studies of targeting of magnetic entities to a desired in vivo location using strong magnets [56,57,58]. Both MRI and MPI can benefit from these targeting strategies to enhance the concentration of imaging agent in a region-of-interest for increased binding probability to targets resulting in better imaging or localization of dose for better therapy. For example, Dames et al. 2007 demonstrated the use of a shaped magnetic tip for targeted delivery of magnetic aerosol droplets to the lung [14] (Figure 1e) and Banura et al. 2017 conducted a similar study with the addition of MPI to image the final biodistribution in the lungs after targeting [13] (Figure 6a). Other than the lungs, permanent magnets have been used to enhance delivery to tumors in other parts of the body. Arami et al. 2017 was able to enhance delivery to a flank tumor using an external permanent magnet affixed to the rodent flank [11].

One limitation of these single-magnet strategies is that targeting is only efficient at regions close to the body surface (Figure 1d). Other than embedding a sufficiently strong magnetic dipole source deep within the body, there is no method to magnetically attract towards an arbitrary point in 3D space. However, the hardware of MPI is able to generate a “repulsive” point at an arbitrary point in 3D space. This is because MPI uses a field-free-point or field-free-line gradient architecture with rapidly increasing magnetic field strength away from the zero-field-region which implies that magnetic material moves towards the edges of the gradient away from the zero-field-point. Magnetic steering is not unique to MPI, and while MRI has been used to steer large magnetic millimeter-sized ferromagnetic beads in vivo before [59], the weaker gradients used in MRI limit the particle size to about 0.2 mm as smaller objects do not have sufficient magnetic mass for MRI gradients to control [60]. The magnetic force F = Ñ(m·B), where Ñ denotes the change of (m·B) per unit distance with units of m^−1^. Assuming a magnetically saturated magnetic moment m (constant) as the object with units of Am^2^, and B as our applied field with base SI units of N A^−1^ m^−1^ (*note Tesla = N A^−1^ m^−1^), then in general the magnetic force F scales linearly with the applied field (gradient) strength. MPI’s 7T/m gradients [12] are much stronger than the 0.045 T/m gradients used in MRI [2] and can provide much larger magnetic forces for targeting. This has resulted in the capability of MPI scanners to remotely steer catheter tips [61], remotely manipulate an iron screw [62], and in theory also steer particles of sub-micrometer scale. Specifically, magnetic catheter steering has seen clinical usage such as the NIOBE^®^ ES Remote Magnetic Navigation (RMN) System (Stereotaxis, St. Louis, MO, USA) albeit with fluoroscopy imaging. In that clinical application, remote magnetic catheter navigation was performed to guide the catheter through the four heart chambers in order to locally perform atrial fibrillation ablation. Over 200 patients were tested, and it was shown that magnetic steering significantly reduced total fluoroscopy time (10.4  ±  6.4 vs. 16.3  ±  10.9 min) and thus lowered radiation dose to the patient when compared with manual pull-wire catheter navigation [63]. Recent preliminary work in the MPI field has shown some promise to completely replace the fluoroscopy aspect of catheter navigation with non-radioactive magnetic imaging by using one MPI “tracer” to mark the catheter tip and a second MPI “tracer” to replace the iodine contrast that shows the blood vessel size, shape, and branching. By distinguishing the magnetic signatures of the two different “tracers”, it enabled interactive magnetic catheter steering with 3D real-time image feedback via “multi-color” MPI [61].

This can be combined with MPI’s relatively high temporal resolution of up to 45 fps [64] to enable scan+steer sequences where an image is taken of a volume within 1/45th of a second every second for real-time image feedback of magnetic targeting while dedicating the 44 other frames to holding the magnetically repulsive point in 3D space. With real-time feedback, this can dynamically target the magnetic material towards an arbitrary region in 3D space despite only using a magnetically repulsive point. Proof-of-concept of this simultaneous imaging and MPI-steering of nanoparticles in Figure 6b–d was demonstrated by Griese et al. 2019 in vitro in a bifurcation flow phantom [65].

As testament to the much stronger gradient strengths used in MPI versus MRI, Griese demonstrated that steering against the flow direction is possible by showing steering of nanoparticles into the arm with a 100% stenosis, although the control experiment showed the flow directs the nanoparticles to the non-stenosed arm when the MPI magnetic force is absent. The concept of seamless switching between “steer” and “image” modes was shown too. With a time ratio of 20:1 for force and imaging mode, the induced magnetic force acts for sufficient durations to maneuver the particles towards the stenosis, although no force is acting on the particles during the short time of the imaging mode [65]. In addition, multiple other studies have shown the feasibility of remote magnetic steering of micron-sized objects or synthetic bacteria in vivo [66,67].

Other than using magnetic forces for targeting, some groups have utilized anaerobic magnetotactic bacteria’s natural tendency to migrate towards hypoxic regions for targeting hypoxic tumor regions [68]. In this case, the targeting depends on the bacteria, but because the bacteria natively produce magnetic crystals, this can be easily imaged with MPI or MRI. However, other groups have used magnetotactic bacteria for magnetic-field controlled manipulation and actuation of micro-objects [69]. Other strategies do not use the magnetic field for attraction forces but mainly for alignment of travel axis. While the bulk of the propulsion comes from micro-turbines or flagella [70,71,72], these micro-swimmers possess a magnetic axis that can be aligned to an external magnetic field for directionality. Unlike MRI having a fixed direction B0 field that limits alignment to the B0 field axis only, MPI’s hardware is well-suited here because the “felt” magnetic field lines can be directed in any arbitrary direction by simply shifting the field-free-point gradient field around since the flux lines just surrounding the field-free-point are directed from every direction towards or away from the point.

## 5. Magnetic Methods for Cancer Therapy in Context of Magnetic Particle Imaging

Magnetic methods for cancer therapy generally fall into a few categories: (1) Hyperthermia methods that raise the temperature of the cancer cells ranging from mild heating to ablative levels via magnetic nanoparticles, (2) Magnetically actuated drug release from cancer-targeted nanocarriers, or (3) Magnetically actuated mechanical disruption of cancer cells by magnetic particles or magnetic micro-/nano-robots. In this section, each category is discussed and the benefits and relevance of Magnetic Particle Imaging towards these methods is explained.

### 5.1. Magnetic Hyperthermia Therapy (MHT)

In brief, MPI unique scanner architecture gives it the potential to be integrated with the alternating magnetic field (AMF) coils used for magnetic hyperthermia, enabling seamless image-guided therapy workflows. Another unique point is that the pre-existing gradients on the MPI scanner can be used to focus magnetic hyperthermia solely at the field-free-point (FFP) or field-free-line (FFL), enabling unprecedented targetable precision at-depth and in a 3D manner. This section explains the background of hyperthermia and recent MPI efforts to synergize with MHT.

The general principle of hyperthermia is based on increasing the temperature of a tissue of interest above 40 degrees Celsius [73,74]. While there are several methods to increase the temperature in hyperthermia, including microwaves, ultrasound, and laser, we focused on radiofrequency magnetic hyperthermia in this article. Magnetic hyperthermia (MH) is a promising cancer therapy that is induced by applying an alternating magnetic field (AMF) of frequencies ranging between 100 kHz and 1 MHz into magnetic nanoparticles targeted in the tumor area [75]. Under such conditions, magnetic nanoparticles act as very local heat sources, which are capable of raising the temperature of cancer tissues and consequently destroying the tumor in a localized and effective way. The heat generated by both superparamagnetic or ferromagnetic nanoparticles is originated from hysteresis losses and is proportional to the area of the hysteresis loop described by the magnetic nanoparticles during the application of the AMF [76,77].

The key advantages of MH are (1) the ability to treat at deeper regions of the body where other surface methods like microwaves, ultrasound, and radiation cannot, (2) negligible energy dose is deposited in healthy tissue en route to the target site as almost all the heat dose comes from the magnetic material on-site, (3) the magnetic material is not consumed by the therapy and allows for multiple treatment sessions per injection, and (4) the thermal dose is externally controlled by the AMF applicator which can compensate for variability in magnetic material accumulation at cancer site to ensure correct thermal dosing [78]. 

The first application of Magnetic Hyperthermia was in 1957 in dogs, where it aimed to treat cancers that had metastasized to the lymph nodes [79,80]. Most of the subsequent studies relied on direct injection of magnetic material into the tumor [81,82] rather than systemic delivery. To address this issue, Ivkov et al., in 2005, utilized monoclonal antibody targeting to cancer tissue [83]. Various other groups used magnetic nanoparticles within cationic liposomes for efficient accumulation into tumors and demonstrated therapeutic effect in rat glioma [84,85,86], melanoma [87,88], and prostate [89] animal tumor models. In recent years, an increasing number of in vivo and in vitro works have been reported in the literature [21,90]. In 2001, Jordan et al. showed the treatment of human solid tumors with MFH [91]. Due to the obtained promising results, several clinical trials have been carried out for the treatment of glioblastoma multiforme and prostate cancer. In 2003, the first phase I clinical trial was performed on 14 patients with glioblastoma multiforme (GBM) at the Charité Hospital in Berlin (MagForce Nanotechnologies) [77,91,92,93]. In 2005, Johannsen et al. reposted the first phase I clinical trial carried out in 10 patients with locally recurrent prostate cancer [94,95,96]. In 2010, MagForce AG obtained European Union Regulatory Approval (10/2011) for its the Nanotherm^®^ therapy and later in 2013 started a clinical study in current gliobastoma with Nanotherm^®^ therapy after receiving approval from the German Federal Institute for Drugs and Medical Devices. Recently, the FDA approved a single-arm study of NanoTherm (R) therapy system for intermediate-risk prostate cancer [97].

Despite all these clinical trials, there are several challenges that need to be addressed. One issue of MH is related to low accumulation of magnetic nanoparticles at the tumor site [98]. In order to achieve an efficient magnetic hyperthermia treatment, the heating efficiency (also known as Specific Absortion Rate (SAR)) of magnetic nanoparticles needs to be as high as possible in order to destroy the cancer with the low amount of magnetic nanoparticle available in the target site. The SAR greatly depends on the physicochemical properties of the nanoparticles such as composition, size, shape, crystallinity, and saturation magnetization [99,100]. Additionally, interparticle magnetic interactions, the interplay between particles and biological systems, and AMF parameters also affect the heating performance of magnetic nanoparticles [101,102].

Currently, different approaches have been proposed in the literature to design magnetic nanoparticles that exhibit high SAR values. Tailoring the shape of the magnetic nanoparticles can provide an effective strategy to increase their heating efficiency. For instance, Guardia et al. showed that the 19 nm iron oxide nanocubes possess very high SAR values (up to 2452 W/g at 29,000 A/m and 520 kHz) compared with spherical particles of similar size [100]. Other promising designs include magnetic vortex nanorings reaching 3000 W/g (at 64,000 A/m and 400 kHz) with demonstrated efficacy in vivo [103]. Some studies also use exchange-coupling between a magnetically hard core and magnetically soft shell to enhance SAR values (3886 W/g at 37,000 A/m and 500 kHz) to an order-of-magnitude greater than conventional iron-oxide nanoparticles, with superior therapeutic effectiveness in mice tumor models over chemotherapeutic drugs [104]. In addition, tuning the arrangements formed by dipolar interactions can also help enhance the heating efficiency of magnetic nanoparticles. Some works in literature have reported that specific arrangements formed by dipolar interaction, like chain-like structures, increase the SAR due to their ability to mechanically orient along the field lines [105,106]. Gandia et al. [107] proved that magnetotactic bacteria of the species M. gryphiswaldense, which internally biomineralized magnetosome chains, give rise to very high SAR values, up to 2400 W/g at 28,000 A/m and 300 kHz. Table 1 provides a summary and key characteristics of these MHT agents.

**Figure 7:**
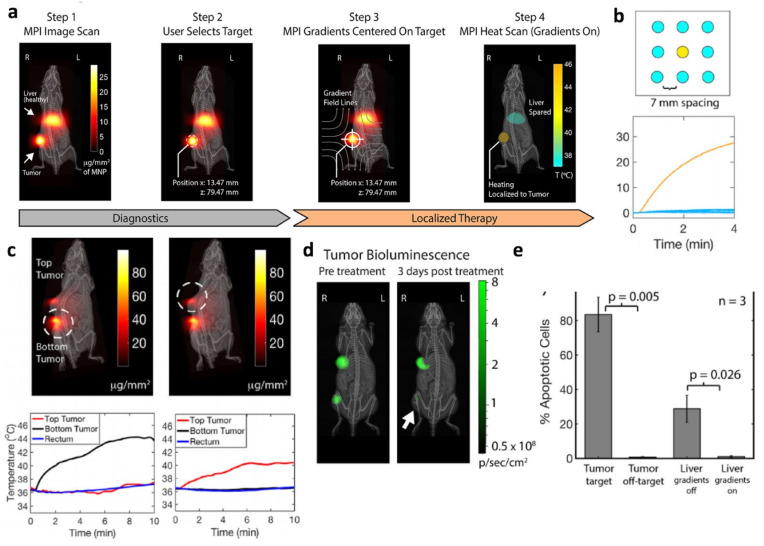
MPI image-guided precision Magnetic Hyperthermia with non-invasive pinpoint heating to 2–7 mm precision at a depth of 4 cm. Adapted with permission from Tay et al. 2018 [15]. Copyright 2018 American Chemical Society. (**a**) Theranostic workflow with MPI image guidance, selection of tumor target, and pinpoint localized heating. (**b**) Precision of at least 7 mm was demonstrated where any user-selected well in a custom 9-well plate can be heated to a 30 degrees increase in temperature with negligible heating in all adjacent targets 7 mm away. (**c**) In vivo results show user-selected precision heating of one of two adjacent tumors to the exclusion of the other. (**d**) Bioluminescence as a viability marker of luc-competent tumor confirms pinpoint therapy of one tumor with negligible impact on off-target tumor. (**e**) Although the mouse liver received a significant nanoparticle dose as shown in a, apoptosis assay showed that MPI hyperthermia (third column) improved precision-to-tumor over conventional hyperthermia (fourth column) which, due to its wide area magnetic excitation, collaterally damaged the liver while treating the tumor. This precision capability can reduce the side-effects of damage to healthy tissue in clinical settings.

Magnetic Particle Imaging provides key benefits for MHT such as image-guidance (Figure 1f), quantitation of magnetic material on-site, which is essential for MHT thermal dose planning, and also the ability to select which magnetic nanoparticles to heat with pinpoint precision as low as a few millimeters [15,108,109,110]. This precision capability is a novel benefit in the field of MHT for cancer therapy. To explain further, consider chemotherapy and radiotherapy, which benefit from a significant differential cytotoxicity between cancerous and healthy cells [111]. Despite this, significant side-effects still exist due to collateral damage to healthy tissue. A similar issue exists for MHT where nanoparticle targeting/trafficking to tumors is not perfect and healthy tissue also accumulate nanoparticles. The additional precision in magnetic excitation enabled by MPI thus greatly mitigates collateral thermal damage to off-target healthy tissues frequently caused by magnetic particles biodistributed to other sites in the body, especially clearance organs such as the liver or spleen. This indirectly increases the therapeutic ratio to allow higher nanoparticle dosage as the side-effects to healthy cells are minimized. This concept is also seen for targeted nanocarriers for drug delivery, where precision of drug release enables higher doses while having lesser side-effects. Details are shown in the next section of this article. Conventional external AMF applicators used in MHT are unable to target magnetic excitation and heating only to the tumor because the long wavelength of the AMF at about ~50 m precludes the possibility of lens-based focusing of the magnetic field at a distance [15]. Other attempts using an array-based synthesization technique were able to project a focal point AMF at a distance of 10 cm but precision remained low with spot sizes of 5 cm [112,113]. Improving the precision to 2.5 cm required exponentially high currents in the kilo-ampere range [113]. In contrast, the mechanism for MPI’s precision heating relies not on “focusing” the AMF into a narrow spot, but rather it suppresses the heating capability of off-target magnetic material by magnetically saturating off-target material so that it cannot respond and get heated by the AMF [15,114,115,116]. This can be achieved by MPI’s field-free-line or field-free-point gradient hardware where the precision linearly scales with the gradient strength [115,116]. For example, in Figure 7b, at a gradient strength of 2.35 T/m, precision of 7 mm was achieved [15]. The field-free-region (zero field point) was simply placed over the target spot, enabling only that point-in-space to respond to AMF while magnetically saturating all other regions in space. Because the hardware for precision targeting exists within the MPI scanner and because the MPI scan at 20 kHz is demonstrated to have zero heating of particles [15], MPI is innately suited for image-guided precision MHT by simply imaging at 20 kHz then switching to a ~300 kHz for gradient-targeted precision MHT. Considering MPI’s fully quantitative imaging of magnetic nanoparticle mass, it is possible to develop the ideal MHT workflow of (1) image, (2) quantitate, (3) dose planning, (4) target positioning, and (5) precision MHT all within a single MPI scanner. This ideal workflow was demonstrated in a rodent cancer model by Tay et al. 2018 (Figure 7a), where the efficacy of precision MHT and mitigation of collateral thermal damage to the liver was validated in vivo [15] (Figure 7c–e).

### 5.2. Magnetically Actuated Drug Release

In brief, MPI can provide image-tracking of the magnetic-labeled drug delivery platform in vivo, ensuring that arrival at the target tumor site has occurred before triggering drug release by magneto-mechanical or magnetic heating in the case of thermosensitive liposomes. Similar to Section 5.1, MPI’s selection field (FFP or FFL) can localize the triggering to only the field-free-region, increasing precision of therapy and further reducing drug side-effects. Finally, MPI images during therapy provide real-time feedback on the extent of drug released from the carrier. This section reviews recent efforts of MPI in the drug delivery field.

Chemotherapy has been one of the mainstays of cancer therapy and there has been much work in developing targeted nanocarriers with controlled release of chemotherapeutic drugs at the tumor to reduce systemic toxicity while maximizing the drug dosage at close proximity to the tumor to improve the therapeutic index [117,118]. Several methods to actuate the release of the chemotherapeutic have been developed and can be widely classified into external stimuli (magnetic, ultrasound, electric field, thermosensitive, UV–vis light, etc.) or endogeneous stimuli (pH-sensitive release, cancer-linked enzyme cleavage reactions, redox reactions, etc.) [119]. Magnetic methods to actuate drug release have several benefits over other methods such as (1) the ability to access deeper regions of the body with no view limitations and (2) the relative safety of magnetic fields compared with other methods for external stimuli that may affect healthy tissue en route to the target [120]. There have been many studies detailed below showing the efficacy of magnetic actuation for controlled chemotherapeutic release. The mechanism relies on a magnetic force to mechanical energy conversion and in many cases there is no detectable temperature rise, although it is also possible to combine both mechanical and MHT heating to doubly trigger release. In 2012, Peiris et al. developed a multi-component iron oxide nanochain with radiofrequency-tunable drug release [121]. The magnetic nanochain efficiently converts magnetic energy from a 10 kHz, 1–50 W external magnetic field into mechanical vibrations that trigger drug release from the attached DOX-loaded liposome. The release rate could be modulated by the operating parameters of the magnetic field. A temperature-sensitive fluorophore attached to the chain acted as a thermometer to verify the absence of local heating. In 2013, Oliveira et al. showed magnetic field triggered drug release (14 mT 750 kHz) from polymersomes, which are notable for their ability to load both hydrophilic and hydrophobic drugs [122]. In 2018, Nardoni et al. used pulsed magnetic fields (20 kHz, 60 A/m) to actuate drug release from high transition-temperature (Tm = 52 °C) magnetoliposomes [123]. The transient increase in membrane permeability upon actuation allowed on-demand drug release while ensuring negligible leakage and safety at all other times. For magneto-thermal mechanisms of drug release, Fuller et al. 2019 demonstrated nanocarriers with a hydrophobic core of superparamagnetic iron oxide nanoparticles that released heat upon AMF to actuate release of drug cargo from a thermoresponsive polymer based on thermally labile Diels-Alder bonds [124].

**Figure 8:**
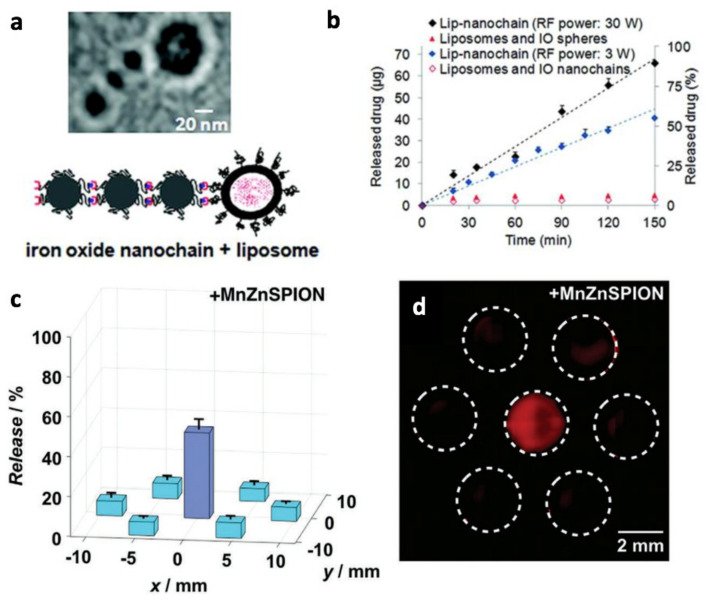
MPI for magnetically-actuated drug release [121]. Top row figures adapted with permission from Peiris et al. ACS Nano (2012) [121]. Copyright 2012 American Chemical Society (**a**) Liposome attached to a nanochain of three SPIONs that mechanically oscillates when exposed to an alternating magnetic field. Rather than using thermo-sensitive release, this work shows the feasibility of mechanical energy for rupturing the attached liposome. (**b**) The extent of drug release can be finely-tuned and controlled by the AMF frequency and power. Bottom row figures adapted with permission from Liu et al. Small (2018) [16]. Copyright 2018 WILEY-VCH Verlag GmbH. (**c**) Magnetic gradients (also termed static gating field in this article) can be used to target drug release to selected locations with 2 mm precision while suppressing release from other neighboring nanocarriers. Since MPI have the strongest magnetic gradients in imaging, the same concept demonstrated in Figure 7 can be replicated here for image-guided targeting of drug release. (**d**) Fluorescence imaging of released DOX from thermosensitive liposomes verifies that only the targeted well triggered drug release.

Magnetic Particle Imaging provides several key benefits for magnetic drug release—(1) Image-guidance and quantitative assessment of nanocarrier accumulation at target tumor site, (2) pinpoint precision of a few millimeters in actuating drug release while suppressing drug release from off-target nanocarriers (Figure 1g), and (3) real-time feedback on the amount of drug released from the magnetic nanocarrier via changes in the magnetic component’s MPI spectrum. Similar to MHT, (1) is crucial for dose planning, especially when the amount of drug release is tunable such as in the study led by Peiris [121] (Figure 8a,b). Benefit (2) works on similar principles to that earlier described for MHT, where the suppression of off-target magnetic entities via magnetic saturation also works to suppress the induced mechanical forces. In other words, the magnetic components are overwhelmed by the background gradient magnetic field and are thus aligned and locked to the directionality of the background magnetic field. MPI’s most unique benefit can be considered to be the real-time feedback on the amount of drug release. The mechanism of this depends on the different microenvironment around the nanoparticles within the nanocarrier as opposed to free nanoparticles after rupture of the nanocarrier. The particles report the change in microenvironment by a quantitative shift in the MPI spectrum. There has been much work in the MPI field to make these “color MPI” algorithms robust and quantitative to microenvironment factors, i.e., viscosity, pH, and inter-molecular binding [38,39,40,41].

Combining benefit (3) with the ability to switch between imaging 20 kHz and actuation ~300 kHz on the same MPI scanner, it is possible to develop the ideal therapy workflow within a single scanner.

**Figure 9:**
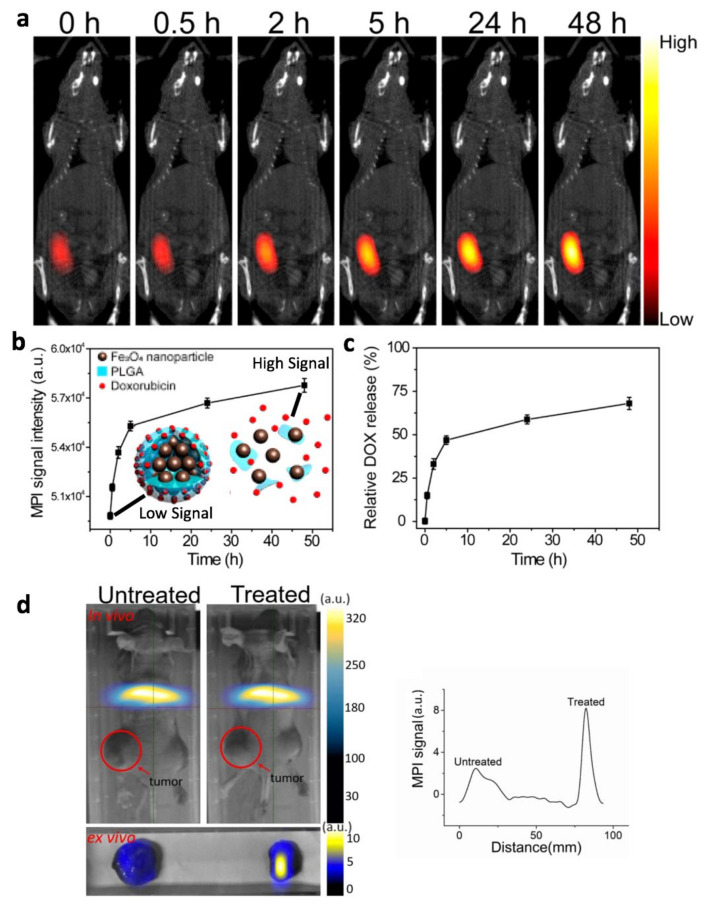
MPI for monitoring of the percentage of drug release from nanocarrier in vivo. Adapted with permission from Zhu et al. Nano Lett (2019) [17]. Copyright 2019 American Chemical Society. MPI enables non-invasive imaging assessment of the extent of drug release via MPI signal differences when SPIONs are encapsulated within a pH-sensitive nanocarrier and after their release together with drug upon nanocarrier rupture. (**a**) In vivo MPI images of nanoparticle-PLGA-Doxorubicin nanocarriers in vivo showing increasing MPI signal over a span of 48 h. (**b**,**c**) Quantification of MPI image intensity and percentage of DOX release from the nanocarrier shows a good correlation between MPI signal intensity and the percentage of release, verifying that the designed nanocarrier works as intended to have low MPI signal pre-release and high MPI signal after the nanoparticles are freed from the nanocarrier together with the Doxorubicin. (**d**) MPI of AnnexinV-SPION that binds to apoptotic cells in mouse xenograft model post-therapy, showing that MPI can evaluate the anti-tumor efficacy of cancer therapy. Figure (**d**) adapted with permission from Liang et al. Phys Med Biol. 2020. [126]. Copyright 2020 by Institute of Physics and Engineering in Medicine. Reproduced by permission of IOP Publishing. All rights reserved.

The ideal workflow of (1) image, (2) quantitate, (3) dose planning, (4) target positioning, (5) precision drug release, and (6) real-time feedback (Figure 1h) on the amount of drug released would be desirable for controlled drug release applications. This workflow is theoretically feasible, although no one group has demonstrated the entirety of this workflow. Separate groups have proof-of-concept studies on each step of the workflow. Maruyama et al. 2016 demonstrated MPI quantitation of magnetic nanocarriers based on a thermoresponsive liposome design [125]. Liu et al. 2018 demonstrated target positioning and precision drug release (Figure 8c,d) at millimeter-scale precision from magnetic nanoliposomes with MPI-like gradient fields (referred to as static gating fields in this paper) [16]. Zhu et al. 2019 used MPI for in vivo quantitative drug release monitoring in tumors of a murine breast cancer model to measure in real-time the amount of drug release [17] (Figure 9a–c). Finally, MPI can be used to monitor apoptosis in tumors post-treatment. Using an apoptosis-specific tracer, MPI can accurately quantify apoptosis as the imaging signal was almost proportional to the number of apoptotic cells [126] (Figure 9d).

### 5.3. Magnetically Actuated Mechanical Disruption of Cancer Cells

In brief, other than magnetic hyperthermia and actuation of drug release, MPI can provide the magnetic energy and control necessary to actuate mechanical disruption of cancer cells. MPI’s unique scanner architecture is well-suited for this because it already has 3-axis drive coils capable of up to 25 kHz and 25 mT field strength to power the magnetic actuation in various directions or to produce rotating magnetic fields. Most importantly, unlike MRI, which has an “always-on” main field forcing a fixed alignment of all magnetic material axes within the MRI scanner, MPI can turn off the electromagnet selection field and allow the drive fields to establish magnetic control of mechanical agents in vivo. This section reviews magneto-mechanical methods for cancer and explains how MPI’s electromagnets can specifically achieve magnetic actuation for these methods.

To introduce the magneto-mechanical approach, we must first note that the magnetic forces incident on magnetic particles can be translated into mechanical energy that directly destroys cancer cells. Creixell et al. 2011 demonstrated that EGFR-targeted magnetic nanoparticles under AMF excitation were able to kill cancer cells (at a 99.9% loss in viability) without a perceptible temperature rise [127]. This runs contrary to the expectation that a temperature rise of up to 43–46 °C is needed to kill the cells under AMF. Because binding and subsequent activation of EGFR is implicated in cancer cell apoptosis, the EGF-nanoparticles without application of AMF already demonstrate some toxicity to the cancer cells. However, the application of AMF significantly increased the toxicity and suggests that magneto-mechanical stimulation of the EGFR via the attached EGF-nanoparticle greatly upregulates the relevant apoptotic pathways. This showcases the feasibility of magneto-mechanical actuation of apoptotic pathways in cancer. Other than EGFR, overactivation of ERK proteins via magnetic particles was also investigated to stop the cancer cell cycle of replication.

Besides mechanical activation of receptor-linked pathways, a more direct method is the mechanical disruption of cellular structures. Externally-bound magnetic particles can compromise cell membrane integrity promoting cell lysis, while internalized magnetic particles cause perturbations in lysozymes leading to enzyme-based cell suicide or damage the cytoskeletal integrity of the cell [128]. Liu et al. 2012 used magnetic carbon nanotubes at 75 mT 16 Hz for magnetoporation of the cancer cell membrane, as measured by increased membrane roughness by Atomic Force Microscopy and Scanning Electron Microscopy [129]. Wong et al. reported similar membrane integrity alteration with magnetic NiFe nanowires at 14 mT 5 Hz via ethidium bromide staining [130]. Domenech et al. showed lysosomal membrane permeabilization in cells that internalized iron oxide magnetic particles with increased the release of proteolytic cathepsin B activity leading to the cancer cell to self-digest [131]. Zhang et al. and Shen et al. reported similar results [132,133], but Master et al. reported negative results where lysosomal disturbance was not observed [134]. Master et al. also targeted the cell cytoskeletal component actin, harnessing the observation that cancer cells are less rigid than healthy cells. The results showed cancer cells were more susceptible to cytoskeletal disruption by actin-targeted magnetic particles under AMF [134]. Additionally, disk-shaped magnetic particles have also been used to magnetomechanical damage cancer cell integrity [135]. The disks can be actuated by an external magnetic field to exercise mechanical force on the cancer cell. Kim et al. used Ni80Fe20 microdisks with magnetic vortex configuration for in vitro experiments with glioma cancer cells. In this work, a 90% of cell death was reported after applying 9 mT and 10–20 Hz during 10 min [136]. Goriena et al. used Ni80Fe20 vortex configuration nanodisks [137] almost ten times smaller than those used by Kim et al. to destroy lung cancer cells [135]. The application of a 10 Hz oscillating magnetic field of 10 mT during 30 min reduced the cell by 30%. Beside disk-shaped magnetic particles with vortex state, perpendicularly magnetized synthetic antiferromagnetic (P-SAF) disks have also been used for cancer treatment through mechanical cell disruption [138].

Magnetic actuation was also demonstrated to be useful in a more macroscopic scale. For example, magnetic microbots at micron-level sizes [139,140,141]. Lee et al. 2020 demonstrated a micron-sized nickel-based magnetic corkscrew that is actuated by an external rotating magnetic field to “corkscrew” itself onto the cancer cell [142]. The microrobot then releases chemotherapeutic drugs after affixing itself to the target cell. Vyskocil et al. 2020 developed Au/Ag/Ni microrobotic scalpels that enter and exit an individual cancer cell and cut the cancer cell under actuation by an external rotating magnetic field [143]. This is relevant to MPI scanners because these rotating magnetic fields can be achieved with the 3-axis electromagnets used to produce the Lissajous trajectory for the FFP. Betal et al. 2018 developed a core-shell magnetoelectric nanorobot that uses DC magnetic gradients for navigation and steering to the target cell. This is relevant to MPI scanners because the DC magnetic gradients needed can be fulfilled by the MPI selection field gradients. The same nanorobot under AMF actuation transforms into a localized electric-pulse generator for targeted cell electroporation. This can directly kill the cancer cells or increase their susceptibility to chemotherapeutics. [144].

As mentioned in previous chapters, MPI systems can provide image-guidance for this type of cancer strategy while offering much stronger AMF (25 mT at 20 kHz) and background gradient capabilities (up to 7 T/m) than MRI systems that typically have a 0.045 T/m gradient and microtesla RF excitation as AMF. While MRI systems can be enhanced with in-bore additions such as the shaped soft-iron core used in dipole-field navigation [145], the tradeoff in image quality due to susceptibility artifacts is usually substantial [60]. As such, MPI’s unique hardware makeup as an imaging modality utilizing strong AMF and strong gradients enables its theranostic capabilities too, leading to better magnetic targeting, control, and actuation for magneto-mechanical strategies to kill cancer cells.

## 6. Safety of MPI and Current Status of Clinical Translation

MPI has comparable safety to MRI, which has been widely recognized as a safe medical imaging modality because it utilizes safe magnetic fields for excitation and signaling. Magnetic fields are non-ionizing and pass through the human body safely without attenuation or any mechanical tissue destruction. There are only two main safety considerations: (1) magnetic stimulation of peripheral nerves causing tingling sensations at the body peripheries when the alternating magnetic field strength is too high and (2) eddy-current induced warming of tissue when the magnetic field is at radio-frequency operating range, which is also known as SAR-related safety limits. For MPI, the drive field operating frequency is relatively low between 1–50 kHz and therefore close to the 42 kHz junction of magnetic stimulation (magstim) safety limits and SAR limit dominance as outlined by the safety study on human volunteers performed by Saritas et al. [146]. This MPI tailored safety study by Saritas et al. is most suitable for MPI’s 20 kHz drive fields that finds no precedence in MRI safety standards. The results showed a limit of 15 mT peak-to-peak for the drive field, which is amenable to MPI imaging-parameters and therefore there are no fundamental safety concerns for MPI.

We can also evaluate MPI safety from the viewpoint of international commission on non-ionizing radiation protection (ICNIRP) standards. For instance, a maximal value of 2.7 Tesla per second for dB/dt (1 Hz–3 kHz, applicable to gradient fields MRI/MPI) was recommended to prevent any magnetostimulation or magnetophosphor effects on patients [147]. MPI’s selection gradient fields, which are shifted by mechanical motion of the patient or by electromagnets at the range of 1 Hz to 3 kHz, stay within the limits proposed. Even for a very strong 7 Tesla per meter selection gradient field, the shift rate can be as high as 0.38 m per second, sufficient to raster the FFP or FFL across a typical clinical FOV dimension of 38 cm in 1 s. For a weaker selection field, this can be proportionally faster. Thus, MPI’s gradient fields have no safety issues under the ICNIRP standards applied to MRI gradient fields.

For Magnetic Hyperthermia, the limits are dominated by the SAR-related safety regime and the safety limits are well-defined by general Atkinson-Brezovich limit (H × f <= 5 × 10^8^ Am^−1^ s^−1^) [148] and the Hergt criterion [149], which is less rigid and tailored to the area of application of the body (H × f <= 5 × 10^9^ Am^−1^ s^−1^). To give examples of existing devices in Johns Hopkins University, Attaluri et al. 2020 constructed a Maxwell-type induction coil prototype for magnetic nanoparticle hyperthermia in phantoms and large animals. The prototype was designed to be scalable to a human-sized system (60 cm diameter) [150]. For hyperthermia devices in Berlin, MagForce AG obtained European Union Regulatory Approval (10/2011) for its Nanotherm^®^ therapy, and clinical studies for glioblastoma therapy were performed in 2013 with this approved instrument. More recently, MagForce received 2020 FDA approval for use in intermediate-risk prostate cancer. These examples and associated references were previously discussed in detail in Section 5.1.

The iron oxide nanoparticles used in MPI have a long history of safe usage in medicine, both as MRI tracer agents and for treating anemia. Iron oxide nanoparticle is considered safe and specific anaphylactic reaction observed when used is often associated with the parenteral formulation (can be made safer) and not the magnetic core that produces the MPI signal [151]. The nanoparticles used in MPI do not contain toxic magnetic elements like cobalt and are entirely iron-oxide based for biocompatibility. Some examples of clinically approved iron oxide are Ferumoxytol (USA) and Ferucarbotran/Resovist^®^ (Japan) and the latter has been shown to work well for MPI [37,152,153]. Regarding clearance, iron oxide nanoparticles are easily assimilated by the liver and spleen and cleared by the hepatobiliary system [154]. Any digested iron from the particles becomes incorporated in the porphyrin rings of hemoglobin [151], replenishing the blood with iron-rich hemoglobin and forming the basis for treating anemia [155].

Although the MPI field has mostly shown preclinical studies, recent work has shown that the imaging technology can be scaled-up to clinical scale. Graeser et al. 2019 showcased a human-sized MPI scanner for brain imaging applications [156]. Mason et al. 2020 showcased an MPI design for clinical intraoperative applications [157].

## 7. Conclusions

Magnetic Particle Imaging is an emerging imaging modality with numerous complementary aspects to the more established MRI in the field of magnetic methods for cancer. Other than direct imaging of tumors, MPI has shown promise to value-add to passive nanocarriers [22] in other aspects such as targeting enhancement, actuating therapy, and post-therapy monitoring. Existing magnetic nanoparticles have mostly been optimized for MRI imaging, but with recent work on optimizing nanoparticles for MPI and heating theranostics, we hope that the capabilities of MPI can be significantly enhanced in the near future by these new classes of nanoparticles. The MPI engineering field has also shown great progress towards clinical translation with recent work showcasing a human head-sized MPI scanner. Overall, MPI has demonstrated its potential in a wide range of applications from tumor imaging to magnetically-actuated in situ drug release. With good compatibility for immunotherapy cell labeling, intrinsic high gradient strengths for magnetic steering and targeting, and finally the capability for spatially precise AMF magnetic heating/actuation, MPI shows great promise as a magnetic platform technology for cancer theranostics. 

## Figures and Tables

**Table 1:** Summary of studies demonstrating MHT agents with high SAR values.

MHT Agent	Characteristics	SAR (W/g)	Ref.
**Iron Oxide Nanocubes**	Size: 19 nm ± 3 nm, Msat: 80 emu/g	2452	[100]
**Magnetic Vortex Nanorings**	Size: 42/70 nm (ID/OD), 50 nm thick K_1:_ 135,000 erg/cc, Msat: 77 emu/g	~3000	[103]
**Core-shell ZnCoFe_2_O_4_ @ZnMnFe_2_O_4_**	Size: 15 nm K: 15,000 J/m^3^ Msat: 125 emu/g	3886	[104]
**Magnetite nanoparticle assembled chains**	Size: 44 nm, sigma = 0.17 Msat: 87 emu/g	4.3-fold SAR w chaining	[105]
**Magnetotactic bacteria M. gryphiswaldense**	Size: 45 nm in 1 micron chain Msat: ~90 emu/g	2400	[107]
